# Genetic Testing for Malignant Hyperthermia Susceptibility—Threading the Needle in the Haystack

**DOI:** 10.3390/genes16111281

**Published:** 2025-10-29

**Authors:** Anjan K. Saha, Teeda Pinyavat

**Affiliations:** Department of Anesthesiology, Columbia University Irving Medical Center, New York, NY 10032, USA; tp2008@cumc.columbia.edu

**Keywords:** malignant hyperthermia, genetic testing, next-generation sequencing, perioperative medicine, patient safety

## Abstract

Malignant hyperthermia (MH) is a rare pharmacogenetic disorder triggered by volatile anesthetics and succinylcholine, most often linked to pathogenic variants in RYR1, CACNA1S, and STAC3. The advent of next-generation sequencing (NGS) has transformed MH diagnostics, offering new opportunities for perioperative risk assessment as caffeine–halothane contracture testing declines. However, challenges remain, including incomplete penetrance, variable pathogenicity of variants, limited access to functional confirmatory testing, and cost. Genetic testing also raises important questions. What is the clinical utility of finding a variant of unknown significance? What are the broader implications of MH susceptibility beyond the operating room? Emerging evidence connects MH susceptibility loci to exertional heat illness (EHI), exertional rhabdomyolysis (ERM), and heat-related mortality, highlighting the need for a broader framework for genetic risk assessment. This review synthesizes historical advances, current consensus, and future directions concerning MH to guide anesthesiologists and perioperative clinicians in leveraging molecular diagnostics for personalized care and improved patient safety.

## 1. Introduction

The completion of the Human Genome Project in 2003 marked a turning point in the medical community’s quest towards developing advanced diagnostics [1,2,3]. Assembly of the initial build, while certainly a feat in and of itself, more importantly laid the groundwork for building tools to mine this biomolecular treasure trove and enhance our understanding of human disease. Next-generation sequencing (NGS) technologies eventually emerged as a high-throughput and high-resolution methodology capable of exponentially expanding our understanding of human diversity as it pertains to health and disease [4,5,6,7,8,9]. Unsurprisingly, the rapid adoption of NGS technologies has accelerated gathering insight into the molecular pathogenesis of various disease processes, in both somatic and germline contexts [10,11,12]. The proliferation of NGS technologies has however produced unanticipated off-target effects on the healthcare ecosystem. Commoditization through direct-to-consumer testing has raised serious questions about intellectual property frameworks, data privacy, and patient education as it pertains to genetic testing [13,14]. The boom-and-bust cycles of commercial entities that offer direct-to-consumer testing further confound our consensus on where NGS technologies fall on the spectrum between asset and liability [15]. While NGS technologies have certainly reframed our expectations of what constitutes an advanced diagnostic, the question remains: what is the value proposition of threading the needles within the vast haystack of the human genome and unearthing them for interrogation through NGS methodologies?

Enter RYR1, one of the many needles that NGS technologies can thread to offer a strong rationale for diagnostic interrogation. RYR1 encodes ryanodine receptor 1 (RYR1), a calcium channel protein expressed primarily in the sarcoplasmic reticulum of skeletal muscle [16,17,18]. The RYR1 channel normally opens in response to a propagated action potential, releasing calcium into the myoplasm, thereby initiating skeletal muscle contraction. Variants in RYR1 are implicated in malignant hyperthermia (MH), a rare hypermetabolic disorder characterized by constitutive activation of the RYR1 channel in response to a subset of anesthetic medications, producing prolonged skeletal muscle contraction [16,18,19,20,21,22]. Signs of an MH trigger include tachycardia, hypercarbia, rigidity refractory to non-depolarizing neuromuscular blockade, and hyperthermia [20,21,22,23,24]. If left untreated with dantrolene, which blocks calcium release through the ryanodine receptor, an MH crisis can lead to hyperkalemia, rhabdomyolysis, disseminated intravascular coagulation (DIC), arrythmias, and death [20,21,22,23,24,25]. As close to 50 million anesthetics are delivered annually in the United States, preventing life-threatening complications through preoperative identification of patients who harbor RYR1 variants appears to be an obvious application for NGS diagnostics [26].

Such an application of NGS diagnostics is however called into question by a few issues: low MH incidence, limitations in genetic testing sensitivity, limited provider education on genetic testing, decommissioning of confirmatory testing (muscle biopsy) centers, and lack of insurance reimbursement. Furthermore, the advent of dantrolene as a mortality reducing “silver bullet” for an MH crisis dampens testing necessity [20,27,28,29]. So, we now contextualize the question asked above: what is the value proposition of leveraging NGS technologies for reducing morbidity and mortality from conditions like MH? In this review, we will delve into the history of MH diagnostics, emphasizing the entrance of NGS methodologies into this domain. We will draw from evidence-based perspectives established by societies in both the United States and Europe who have protocolized our understanding of the genetic underpinnings that drive MH. We will pay special attention to the nonsurgical implications of a genetic diagnosis of MH, with a discussion of evolving epidemiologic trends that have potential links to MH risk loci. Most importantly, our review will facilitate educating the medical community on leveraging the latest developments in advanced diagnostics to genetically assess preoperative risk of life-threatening conditions like MH.

## 2. Diagnostic Testing for MH

Though fever, convulsions, and sudden death from anesthesia had been documented as early as the days of ether and chloroform administration in the early 1900s, the first breakthroughs in understanding the heritable nature of these reports arose in 1960 [21,30]. Michael Denborough, an anesthesiologist in Melbourne, Australia, described the case of a young man with a combined fracture of the tibia and fibula expressing heightened concern for receiving a general anesthetic due to a significant family history of morbidity and mortality from ether administration. During a halothane based general anesthetic, the young man displayed signs of hypermetabolic derangement including tachycardia, hypotension, and hyperthermia [30]. Early recognition, discontinuation of halothane administration, supportive care, and expeditious completion of surgery fortunately facilitated an uneventful recovery. Subsequent evaluation of the proband’s pedigree revealed autosomal dominant inheritance of death from anesthesia, with all deceased relatives having exhibited a similar constellation of symptoms after receiving ether or ethyl chloride.

The subsequent six decades spawned active community engagement and rigorous investigations into triggering agents, diagnostic testing strategies, genetic underpinnings, disease epidemiology, and bedside therapeutics to guide management of what became known as malignant hyperthermia (MH). Professional societies like the Malignant Hyperthermia Association of the United States (MHAUS) and European Malignant Hyperthermia Group (EMHG) were established to raise awareness and drive progress on disease characterization [31,32,33,34,35]. Halogenated hydrocarbons (volatile anesthetics) and succinylcholine were added to the list of agents known to trigger an MH crisis [36,37,38,39]. The caffeine halothane contracture test (CHCT), a contracture assessment of biopsied muscle tissue, became the gold standard for diagnosing MH, a significant advance from the utilization of clinical signs and basic laboratory testing alone [21,40,41]. *RYR1* alterations were identified as the first genetic loci implicated in the inheritance and pathogenesis of the disease, making up the vast majority of MH cases (50–60%) [16,17,18,42]. While the prevalence of *RYR* drivers is as high as 1:800, only 1:10,000 to 1:150,000 anesthetics manifests as an MH crisis [43,44,45]. Yet if untreated, mortality from MH is as high as 80% [46]. The Food and Drug Administration’s (FDA) approval of dantrolene in 1979, along with widespread education and regulation centered on MH recognition and treatment, shifted the mortality curve dramatically [27,28,29,47]. Today, estimated mortality from MH ranges from 3 to 10% in high- and middle-income countries [27,46].

Intriguingly, the identification of *RYR1* alterations as pathogenic drivers of MH came *after* the very measurable impact of dantrolene therapy on disease morbidity and mortality. While molecular genetics did not necessarily inform the development and approval of dantrolene for MH, it has certainly provided necessary context to further inform patients and providers about the pharmacogenomic drivers of disease. At the turn of the century, *CACNA1S* was identified as another locus that conferred MH susceptibility (MHS), also inherited in an autosomal dominant fashion [48]. *CACNA1S* encodes a calcium channel in the transverse tubules that mechanistically supports the function of the ryanodine receptor. Unsurprisingly, a subset of alterations in *CACNA1S* manifest similar phenotypes as *RYR1* variants in the presence of triggering agents, though at a much less frequent rate than *RYR1* (1%) [42,48]. Similarly, *STAC3*, a gene that encodes a structural protein integral for skeletal muscle contraction, has also been linked to MHS, though inherited in an autosomal recessive fashion and at the lowest rate of the three known susceptibility genes (<1%) [42,49,50]. These loci, in addition to a series of emerging susceptibility loci (*CASQ1*, *ATP2A1*, *ASPH*, *HRC*, and *TRPV1*), have now been included in NGS testing panels that are offered to aid in diagnosing patients with high suspicion of having triggered an MH episode [51,52,53]. With the progressive closure of testing facilities equipped to perform the CHCT, the utility of NGS technologies for MH diagnostics is likely to grow [54,55].

## 3. Translational Genetics of MH

While the growing demand for NGS technologies can certainly offset the decommissioning of CHCT testing centers from a diagnostic standpoint, the practical limitations of NGS methodologies have major implications on bedside decision making for providers. Moreover, the genetic principles that drive the phenotypic presentation of disease add nuance to assessing the viability of deploying NGS technologies in the perioperative arena. Both the practical and theoretical considerations behind widespread use of NGS technologies can be summarized by framing the discussion from the perspective of two foundational concepts in clinical genetics: pathogenicity and penetrance.

Pathogenicity is defined as the inherent capacity of a genetic alteration to disrupt gene or protein function, thereby leading to a state of disease [56]. As it pertains to MH, not all variants in susceptibility loci exhibit equal pathogenicity. In other words, not every alteration in *RYR1*, *CACNA1S*, or *STAC3* equally disrupts gene or protein function enough to manifest as an MH episode in response to a triggering anesthetic [57]. As such, various national and international consortia have devised classification systems to risk stratify known variants in MHS loci [32,50,52,58,59,60,61]. These pathogenicity assessments not only guide laboratory interpretation but also serve as decision-support tools that link molecular data to anesthetic risk management. By characterizing variants as pathogenic, likely pathogenic, benign, likely benign, or a variant of unknown significance (VUS), bedside providers can triage test results to determine the safety margin of using triggering agents if they are otherwise indicated. Genetic triage however has limited utility in situations where a patient tests negative for known variants; a negative test does not necessarily mean that a patient is free of pathogenic mutations that would be identified by way of methodologies with more comprehensive genomic coverage [54]. Yet wider coverage may increase the false positive rate by identifying variants outside of known susceptibility loci that play no mechanistic role in MH pathogenesis [62]. Providers thus have to weigh both the reported susceptibility loci and the testing methodology when interpreting NGS test results at the bedside. For example, a patient with a family history of MH who tests positive for a pathogenic *RYR1* variant by both panel testing and whole genome sequencing (WGS) should be treated as being MH susceptible, but a patient with a family history of heat intolerance who has a negative panel test but flags positive for a VUS outside of known susceptibility loci by WGS requires more nuanced consideration.

Even more harrowing than interpreting pathogenicity is predicting penetrance of a pathogenic variant. Penetrance is defined as the proportion of patients with a known pathogenic variant who exhibit the associated disease phenotype [63,64]. It is widely recognized that known pathogenic/likely pathogenic variants for MHS display incomplete penetrance [51,54]. Often, an MH susceptible patient exposed to a triggering anesthetic will not display signs of an MH episode, even over several encounters, but are eventually found to have a pathogenic/likely pathogenic variant through NGS testing after eventually succumbing to an MH event [54]. At present, there are no known predictive factors that can aid bedside providers in determining whether a carrier of a pathogenic/likely pathogenic variant will display signs of MH from a triggering anesthetic. It is exceedingly difficult to assess likelihood of manifesting an MH crisis in the absence of triggering agents. The safest strategy during an encounter with a patient who reports a positive family history of MH or is incidentally found to have a pathogenic/likely pathogenic variant from genetic testing is to avoid triggering agents (volatile anesthetics and succinylcholine), also known as “running a non-triggering anesthetic” [20,46,65]. This traditionally involves total intravenous anesthesia (TIVA) as an alternative to volatile inhalational agents, supplemented with non-depolarizing neuromuscular blockade (rocuronium, vecuronium, etc.) as an alternative to succinylcholine.

Although variable pathogenicity and incomplete penetrance complicate interpretation, NGS testing for MHS when applied with appropriate patient selection, careful test choice, and clinical geneticist involvement, has markedly improved the ability to prevent fatal MH episodes. The specific NGS methodology employed remains a critical determinant of the diagnostic yield and clinical utility of testing [23,51]. Methods with wider coverage like WGS are expensive and subject to higher false positive rates, whereas cheaper, more targeted methods may have higher false negative rates [62,66,67]. VUS’s would also present providers with information that may not have otherwise had clinical consequences. Insurance coverage for NGS screening in the preoperative setting is also not standardized currently. Nevertheless, the variation in pathogenicity and penetrance for a disease with high morbidity (and high mortality if unrecognized and untreated) provides a possible runway to justify perioperative NGS screening to ensure safety and personalized care.

## 4. Beyond the Operating Theater

Because assessing pathogenicity and penetrance presents providers with the difficult task of predicting the likelihood of an MH crisis, it is worth paying consideration to emerging nonsurgical epidemiologic trends that may prove useful in providing context to preoperative risk assessments involving NGS technologies. A recent retrospective study highlighted an alarming rise in heat-related deaths from 1999 to 2023 in the United States [68]. This rise was most prominent from 2016 to 2023. While the authors of the study placed emphasis on the impact of rising global temperatures on the increasing prevalence of heat-related deaths, they also noted that lack of data from vulnerable subgroups may have introduced bias into the study. These vulnerable subgroups, while not explicitly stated, may include individuals with genetic predisposition to hypermetabolic syndromes that are unmasked in the presence of environmental triggers. This could include patients who have known variants in MHS loci, where a triggering anesthetic would constitute an environmental trigger.

With the prevalence of *RYR1* alterations being as high as 1:800, the results from this retrospective study come as no surprise. The link between MHS and exertional heat illnesses (EHI) and exertional rhabdomyolysis (ERM) has indeed been previously reported and is well established [20,69,70,71,72,73,74]. Variants in MH susceptibility loci have known association with a predisposition for EHI/ERM. Athletes with traumatic muscle contractures have been shown to benefit from dantrolene, the medication that has drastically reduced mortality from MH [75]. Personal history of MH is a medically disqualifying condition for service in the United States military, owing to the risk of EHI/ERM if deployed into environmentally strenuous circumstances [51,76,77].

The links between MHS and EHI/ERM provide additional validation for conducting preoperative NGS screening [55]. The preoperative arena can be leveraged not only for risk stratification for surgery, but as a waypoint for screening for EHI/ERM. Such screening, however, has implications for patients with documented heat intolerance, athletes, and military enlistees. In addition to previously discussed considerations surrounding testing methodology and test performance, offering wide-scale preoperative NGS testing would require clinical capacity to offer care to family members of patients who test positive for MHS loci. Genetic counseling services would need to be made available for instances where results from testing impact the livelihoods of patients and their families beyond the operating room [51]. The role of medical geneticists is also important to consider, given the domain expertise they can provide to support intraoperative providers who are not as familiar with testing methodologies and the implications of a positive test [51,52].

While mechanistic links between MH, EHI, and ERM are well established through variants in RYR1 and related loci, variable expressivity and limited penetrance suggest that only a minority of those with exertional syndromes carry MHS pathogenic variants. Careful consideration of clinical context, family history, anesthetic exposure, and functional testing will remain crucial in interpreting NGS results for RYR1 and allied genes, given the complex interplay of polygenic, epigenetic, and environmental factors that likely underlie ERM and EHI, except in rare monogenic instances. While the benefits of conducting wide-scale preoperative NGS screening for MHS are substantial, foresight will be necessary to ensure that the implementation of screening is patient centered while attempting to thread the needles in the haystack.

## 5. Final Thoughts

As the cost of NGS testing continues to fall, educating providers who encounter heritable syndromes like MH will be an essential aspect of ensuring a smooth transition to a “post-genomic” era of perioperative medicine [54]. A comprehensive understanding of the genetic drivers of disease, available testing modalities (and their interpretations), and the nonsurgical implications of conducting NGS testing will best position bedside practitioners for favorable outcomes. As it pertains to MH, evolving epidemiologic trends due to environmental changes remind us that emerging information on population disease profiles can profoundly impact previously held dogmas regarding the value of routine screening. Professional societies with expertise in MH pathogenicity and penetrance will be essential to devising algorithms that protocolize the shifting epidemiologic landscape. Most importantly, cost-effectiveness analyses of performing preoperative NGS screening for MH will be a critical component of obtaining buy-in from insurance companies who would ultimately underwrite the cost of testing.

MHS loci, however, comprise only a handful of the needles within the haystack of the human genome that can be threaded through NGS testing methodologies. Pseudocholinesterase deficiency, Factor V Leiden, and mitochondrial disorders represent additional examples of pharmacogenomic conditions that like MH can be identified by way of NGS screening [78]. These syndromes all carry increased risk of perioperative complications that impact morbidity and mortality. It is also important to consider cytochrome P450 polymorphisms, which can alter drug metabolism, as part of the broader spectrum of pharmacogenomic disorders that may affect patient outcomes. Screening for “perioperative risk loci” collectively could thus enhance the clinical utility of routine NGS testing more so than screening for any one of these pharmacogenomic disorders individually. The emergence of technologies like cell-free DNA interrogation produces a compelling case for conducting risk loci testing, particularly if done in parallel to more routine assessments of metabolic panels and blood counts.

While NGS testing has shifted our expectations of what constitutes an advanced diagnostic, deployment at scale for perioperative risk stratification necessitates careful attention to limit unintended consequences. With annual surgical volume expected to rise over the coming decade, regulatory guard rails will be vital to ensuring that NGS repositories can withstand legal scrutiny from the perspective of maintaining patient privacy. Nevertheless, these biomolecular treasure troves are immensely valuable tools that have enhanced our understanding of human disease since the completion of the Human Genome Project. Continued investment in these tools will be an indispensable component in our quest towards enhancing patient safety and quality of care.

## Data Availability

No new data were created or analyzed in this study. Data sharing is not applicable to this article.
